# The Effect of Superficial Heat–Cold Application on the Sleep Quality of Patients With Restless Leg Syndrome: A Systematic Review and Meta‐Analysis

**DOI:** 10.1002/nop2.70080

**Published:** 2024-11-11

**Authors:** Mohammad Mehdi Mohammadi, Maryam Ahmadi, Ali Akbar Vaisi Raygani

**Affiliations:** ^1^ Department of Medical‐Surgical Nursing, School of Nursing and Midwifery Kermanshah University of Medical Sciences Kermanshah Iran; ^2^ Kermanshah University of Medical Sciences Kermanshah Iran

**Keywords:** cryotherapy, meta‐analysis, nursing, restless legs syndrome, sleep quality, systematic review, thermotherapy

## Abstract

**Aim:**

The present study was conducted to determine the effect of the superficial heat–cold application on the sleep quality of patients with restless leg syndrome.

**Design:**

This study was a systematic review and meta‐analysis.

**Methods:**

In the present study, the electronic databases Scopus, ProQuest, Web of Science, PubMed, SID and Google Scholar were searched from their inception to September 2023. The quality of included studies was evaluated through the Cochrane Collaboration's Risk of Bias Tool, and finally, a meta‐analysis was conducted by calculating standardised mean differences (SMDs).

**Results:**

The meta‐analysis results revealed that superficial heat–cold application improved sleep quality in patients with RLS (SMD = 0.685, 95% CI: 0.421–0.950). The meta‐regression results showed that as the temperature increased, the intervention was more effective in improving sleep quality (*β* = 0.0182, 95% CI: 0.0096–0.0268, *p* < 0.05). Moreover, the effectiveness of the intervention in improving the sleep quality of patients with RLS reduced significantly as the duration of intervention in each session (*β* = −0.031, 95% CI: −0.059 to −0.001, *p* < 0.05) as well as participants' age increased (*β* = −0.013, 95% CI: −0.024 to −0.001, *p* = 0.0259).

**Patient or Public Contribution:**

This research showed that superficial heat–cold application had the capability to improve the sleep quality of patients with restless leg syndrome. In addition, in this study, settings were suggested according to which the maximum effectiveness of the intervention could be achieved.

## 1 Introduction

Restless legs syndrome (RLS), scientifically known as Willis‐Ekbom (WED), is a sleep‐related movement disorder characterised by an often irritating or disturbing urge to move the legs (Guffey et al. 2016; Perez‐Diaz et al. 2011). In studies conducted mainly on European and North American populations, RLS was prevalent in 5%–15% of adults (Ohayon et al. 2016; Ondo 2022).

An evident symptom of RLS is an unpleasant or uncomfortable desire to move the legs. This symptom appears during periods of inactivity, commonly in the evening, is temporarily relieved by movement and is usually felt deep inside the legs, often between the knees and ankles. Patients find this sensation difficult to describe; however, standard terms used by individuals with RLS to refer to the symptoms include ‘need to move’, ‘crawling’, ‘tingling’, ‘restlessness’, ‘cramping’, ‘creeping’, ‘pulling’, ‘tension’, ‘discomfort’, ‘pain’ and ‘itching’ (Guo et al. 2017; Oskarsson, Wåhlin‐Larsson, and Ulfberg 2014).

Since symptoms of the syndrome deteriorate at nighttime, patients with RLS may have trouble falling asleep or waking up several times during sleep (Massey and Robertson 2020; Sahli et al. 2017). In other words, this syndrome leads to impaired sleep quality, resulting in irritability, sleep deprivation, fatigue during the day, weakening of daytime functioning, excessive daytime drowsiness, sadness, depression and anxiety (Alhothaly et al. 2021; Habibzadeh, Lazari, and Ghanei Gheshlagh 2013; You 2019).

Pharmacological and nonpharmacological approaches are among the methods to improve sleep quality and symptoms in patients with RLS (Guffey et al. 2016). In this regard, the common drug categories include anticonvulsants, opioids, dopamine agonists, benzodiazepines and medications such as clonidine (Aurora et al. 2012). These drugs cause side effects such as disorders of blood pressure regulation, drowsiness, RLS symptom intensification in the morning, RLS early onset at night, drug tolerance, recurrent symptoms, increased severity of the syndrome compared to the period before medication use and the spread of the syndrome symptoms to the arms (Ferini‐Strambi et al. 2008; Winkelman and Johnston 2004).

More recommendations have been made on non‐pharmacological approaches due to their limited adverse effects compared to the pharmacological approach (Mitchell 2010). Superficial heat–cold application is a non‐pharmacological approach involving applying heat or cold to change the skin and soft tissue temperature. This therapeutic method is based on superficial techniques (such as heat or cold pack [grain]) in which conduction or convection mechanisms are used. This therapeutic method is more affordable and safer than deep techniques (such as diathermy, laser therapy and ultrasound) based on the conversion mechanism (Freiwald et al. 2021).

Superficial heat application can be considered one of the prevalent treatments for relieving the pain of musculoskeletal origin. This method increases the skin temperature, thus increasing blood circulation to provide more nutrients and oxygen to the cells. Additionally, the superficial cold application increases the leukocytes, decreases histamine and strengthens the immune system. Evidence suggests that this method has antioxidant effects and affects plasma norepinephrine, resulting in an increased neurotransmitter release (Jafarimanesh, Vakilian, and Mobasseri 2020a, 2020b).

Historically, nurses have had a long‐standing relationship with superficial heat–cold application. Nasiriani reports in his article that nurses have traditionally utilised heat and cold to relieve diseases; however, it is necessary to prove the effectiveness of these interventions through valid scientific evidence (Nasiriani and Eftekhari 2016). Recent studies have published findings on the effects of superficial heat–cold on sleep quality in patients with RLS. However, to date, a definitive result on the effect of this intervention on RLS patients' sleep quality has not been published. Therefore, this systematic review and meta‐analysis attempted to combine the results of clinical trial studies and publish a conclusion and practice guide for healthcare providers. Since no systematic review and meta‐analysis have been conducted in this field, the present study was carried out to determine the effect of the superficial heat–cold application on the sleep quality of patients with RLS.

## 2 Methods

### 2.1 Search Strategy

In this research, the Preferred Reporting Items for Systematic Reviews and Meta‐analyses (PRISMA) statement was considered as a practical guide (Liberati et al. 2009). The present study is based on the electronic databases Google Scholar, Web of Science, ProQuest, Scopus, PubMed and SID from inception to September 2023. In addition, the search for similar studies was performed manually by checking the references of the found studies.

Moreover, the databases: ClinicalTrials.gov, EU register, Australia and New Zealand (ANZCTR), ISRCTN Registry, Japan Registry of Clinical Trials (JRCT), Cochrane Library, Iranian Registry of Clinical Trials (IRCT) and The Japan Primary Registries Network (JPRN) were searched to find the registered trials. After finding a clinical trial in the above databases, in the first step, it was investigated whether the article related to that clinical trial was published or not. If the article was not published, the corresponding author was contacted through email, and the required information was obtained.

Persian and English languages were used in searching for studies. The keywords used in the search included (‘Cryotherapy’ OR ‘thermotherapy’ OR ‘temperature’ OR ‘heat wrap’ OR ‘cold wrap’ OR ‘water bottles’ OR ‘heat pack’ OR ‘cold pack’ OR ‘hot poultices’ OR ‘cold poultices’ OR ‘heat lamp’ OR ‘electric heat pads’ OR ‘hydrotherapy’ OR ‘hot water baths’ OR ‘cold water baths’ OR stream OR sauna) AND (‘Sleep quality’ OR ‘Sleep Initiation and Maintenance Disorders’ OR ‘sleeplessness’ OR ‘insomnia’ OR ‘sleep maintenance’ OR ‘sleep disturbance’ OR ‘sleep problem’ OR ‘sleep’ OR ‘sleep promotion’) and their Persian equivalents. In order to write the search strategy, the keywords ‘intervention’ and ‘outcome’ were used in the intended strategy. Keywords were selected for intervention (superficial heat–cold) and outcome (sleep quality) based on MeSH and free text terms. For complete information about the study search strategy, see the appendix (Appendix 1).

### 2.2 Inclusion and Exclusion Criteria

The inclusion criteria were as follows:

#### 2.2.1 Study Type

Clinical trials and quasi‐experimental studies.

#### 2.2.2 Study Population

Patients with RLS meeting the following four criteria: (1) repeated movement along with unpleasant sensation in the legs, (2) temporary relief of unpleasant symptoms by moving the legs, (3) onset or exacerbation of symptoms after resting or inactivity of the legs, and (4) the onset or aggravation of symptoms in the evening or at nighttime.

#### 2.2.3 Intervention

Superficial heat–cold used as an intervention in the following way: heat or cold used superficially, and each of the methods' heat or cold wrap (wearable)’, ‘hot or cold water bottles,’ ‘hot or cold stone therapy,’ ‘heat or cold pack (grain),’ ‘hot or cold poultices’, ‘heat lamp,’ ‘electric heat pads,’ ‘hydrotherapy,’ ‘hot or cold water baths’ and ‘stream/sauna’ used between −100°C and +100°C.

#### 2.2.4 Outcome

Researches quantitatively reporting sleep quality as an outcome. Exclusion criteria included non‐human studies and studies that combined superficial heat–cold application with other interventions effective on RLS.

### 2.3 Study Selection

The search for studies in each database was firstly performed based on the search strategy defined for each database. Afterward, registered clinical trials, theses, dissertations, conference proceedings and bibliographies or a list of references were searched. Then, duplicate studies were removed manually as well as using Endnote software. The remaining studies entered the initial screening stage, during which the title and abstract of each study were reviewed to assess the eligibility of the study. After the completion of the initial screening stage, the secondary screening stage began. In this stage, the full text of the studies recognised as relevant based on their title and abstract were reviewed, and the eligible articles were included in the study. In all stages, the search was conducted independently by two researchers (MMM and MA). It should be noted that in case of disagreement between the two researchers, the third researcher (AVR) mediated to resolve the conflict.

### 2.4 Evaluating the Quality of Studies

The Cochrane Collaboration's Risk of Bias Tool was used to assess the quality of studies (Higgins et al. 2011). To this end, two independent researchers rated the bias risk based on the low/high/unclear scoring scale (Table 1).

**TABLE 1:** Risk of bias in included studies^a^.

Study	Selection bias		Patient blinding	Assessor blinding	Incomplete outcome data	Selective outcome reporting
	Random sequence generation	Allocation concealment				
Happe et al. (2016)	L	L	L	L	L	L
Jafarimanesh (2017)	L	L	U	L	L	L
Jafarimanesh, Vakilian, and Mobasseri (2020a)	L	L	U	L	L	L
Park, Ambrogi, and Hade (2020)	L	L	U	L	L	U
Eftekhari et al. (2022)	NA	NA	U	U	L	L
Azizkhani et al. (2022)	L	U	U	U	L	L
Shamekh et al. (2022)	NA	NA	U	U	L	L

Abbreviations: H, high risk of bias; L, low risk of bias; NA, not applicable; U, unclear (uncertain risk of bias).

^a^ Domains of quality assessment based on the Cochrane tools for assessing risk of bias.

Random Sequence Generation was performed in two studies using a computer to create a randomisation list (Azizkhani et al. 2022; Happe et al. 2016). In three other studies, the random block method was used as the basis (Jafarimanesh, Vakilian, and Mobasseri 2020a, 2020b; Park, Ambrogi, and Hade 2020).

In one study, allocation concealment was implemented based on a sealed envelope (Happe et al. 2016). In this regard, one of the studies obtained assistance from a central unit (Park, Ambrogi, and Hade 2020). Nevertheless, in other studies, there was no definitive mention of allocation concealment (Azizkhani et al. 2022; Eftekhari, Nasiriani, and Baghian 2022; Jafarimanesh, Vakilian, and Mobasseri 2020a, 2020b; Shamekh et al. 2022).

Blinding patients was clearly reported in only one study (Happe et al. 2016). Moreover, there were no incomplete outcome data in reviewed studies due to reporting number, nature and management of incomplete outcome data (Azizkhani et al. 2022; Eftekhari, Nasiriani, and Baghian 2022; Happe et al. 2016; Jafarimanesh, Vakilian, and Mobasseri 2020a, 2020b; Park, Ambrogi, and Hade 2020; Shamekh et al. 2022). Other information is presented in Table 1.

### 2.5 Data Extraction

Two researchers (MMM and MA) performed data extraction independently. To this end, the data extraction form was used, which included the author's name, the name of the country, the year of publication, samples' age, the study design, the duration of the intervention, the sample size, the temperature of the intervention, the population, the control group, the outcome and measuring tools.

### 2.6 Statistical Analysis

The present study used Comprehensive Meta‐Analysis software (CMA, and New England, NJ, USA) to conduct a meta‐analysis. The effect size was calculated based on the standardised mean difference with a 95% confidence interval. The random effects model was the basis of the calculations. In order to analyse the heterogeneity of the studies, the *I* ^2^ value was used. Subgroup analysis and meta‐regression were also used in order to further investigate the source of heterogeneity. Accordingly, the included studies were assigned to subgroups based on the country, underlying clinical conditions, study type and the control group. In order to conduct meta‐regression, the duration of the intervention, the year of publication, the number of treatment sessions, participants' age and the temperature of the intervention (Celsius) were considered moderator variables. It is noteworthy that pre–post correlation or standard deviation for changes (SD_change_) was reported in none of the included studies. Therefore, the pre–post correlation was calculated based on the sensitivity measures. In other words, the meta‐analysis was run independently with zero, 0.5 and 0.99 correlations, and in all cases, the overall result of the calculations was estimated to be similar. Consequently, the researchers finally estimated the pre‐post correlation at 0.5.

Publication bias was performed based on two methods: Funnel Plot and Egger's test. In this study, sensitivity analysis was performed in order to check the robustness of the study results.

## 3 Results

### 3.1 Study Selection

**FIGURE 1:**
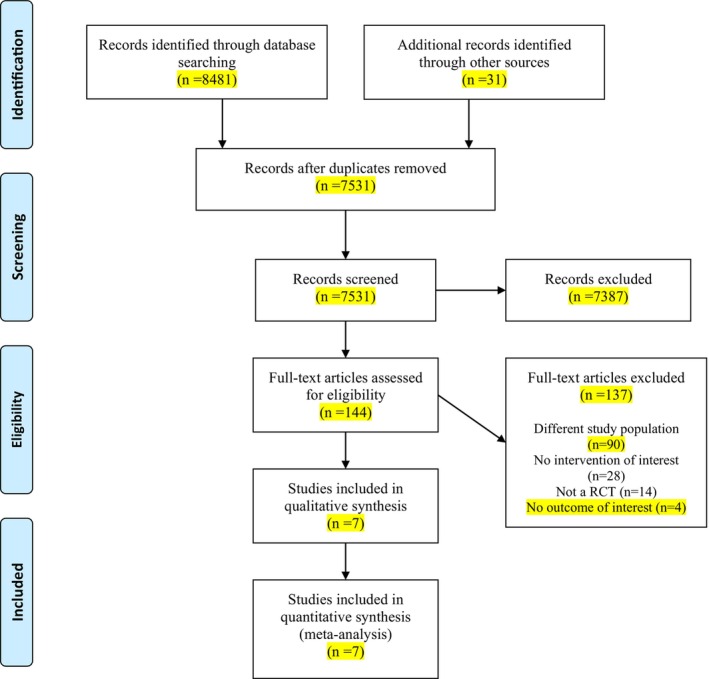
PRISMA flow diagram of the present study.

Searching electronic databases, 8481 studies were initially found. In addition to electronic databases, other sources including theses and dissertations, conference proceedings, registered clinical trials, and bibliographies or the list of references were searched through which 31 studies were found (7 studies in clinical trial registration databases and 24 studies through manual search of reference of studies). Afterward, duplicate studies (a total of 981 studies) were removed, and 7531 studies remained. Subsequently, in the initial screening stage, the studies were reviewed regarding title and abstract, and thus, 144 studies were selected. In the following step, through secondary screening, the full text of the studies was examined; 7 studies were considered eligible, and 137 were excluded (Appendix 2). Finally, the present study was conducted on seven studies, and qualitative and quantitative analyses were performed based on them (Figure 1).

### 3.2 Study Characteristics

Seven studies were included, two of which were clinical trials (Happe et al. 2016; Park, Ambrogi, and Hade 2020), and five were quasi‐experimental (Azizkhani et al. 2022; Eftekhari, Nasiriani, and Baghian 2022; Jafarimanesh, Vakilian, and Mobasseri 2020a, 2020b; Shamekh et al. 2022). The publication year of the studies under investigation ranged between 2016 and 2022. The total number of samples in these studies was 259 individuals, whose mean age was estimated to be 49.39 years. The target studies were conducted in Germany (Happe et al. 2016), the United States of America (Park, Ambrogi, and Hade 2020), Iran (Azizkhani et al. 2022; Eftekhari, Nasiriani, and Baghian 2022; Jafarimanesh, Vakilian, and Mobasseri 2020a, 2020b) and Egypt (Shamekh et al. 2022). One study (Jafarimanesh, Vakilian, and Mobasseri 2020a, 2020b) was printed in the Persian language, and five studies (Azizkhani et al. 2022; Eftekhari, Nasiriani, and Baghian 2022; Happe et al. 2016; Park, Ambrogi, and Hade 2020; Shamekh et al. 2022) were in English (Table 2).

**TABLE 2:** Characteristics of included studies.

Study (years)	Country	Design of study	(Total duration of treatment) (Weeks) (Sessions) (min/session)	Intervention temperature	Sample size (EG, CG)	Population (Age)	Control group	Outcomes	Instrument
Happe et al. (2016)	Germany	Clinical trial	(84 min) (2) (14) (6)	≃ −17°C	EG:12 CG:11	Patients with idiopathic RLS (EG: 64.3 ± 8.6 CG: 61.6 ± 11)	Sham group	Sleep quality	DGSM
Jafarimanesh (2017)	Iran	Quasi‐experimental	**(**140 min) (2) (14) (10)	40–45 ≃ 42.5°C	EG:37	Pregnant women with RLS (EG: 28.01 ± 5.48)	Without control group	Sleep quality	PSQI
Jafarimanesh, Vakilian, and Mobasseri (2020a)	Iran	Quasi‐experimental	**(**140 min) (2) (14) (10)	20–25 ≃ 22.5°C	EG:40	Pregnant women with RLS (EG: 27.58 ± 4.88)	Without control group	Sleep quality	PSQI
Park, Ambrogi, and Hade (2020)	United States	Clinical trial	(840 min) (4) (28) (30)	≃ 58.8°C	EG:6 CG:7	Patients with idiopathic RLS (EG: 61.8 ± 6.2 CG: 54.2 ± 10.4)	No intervention in control group	Sleep quality	Medical outcomes sleep study scale
Eftekhari et al. (2022)	Iran	Quasi‐experimental	(240 min) (4) (12) (20)	15°C	EG:36	Haemodialysis Patients with RLS (EG: 60.86 ± 16.43)	Without control group	Sleep quality	PSQI
Azizkhani et al. (2022)	Iran	Quasi‐experimental	(600 min) (4) (30) (20)	40–43 ≃ 41.5°C	EG:40 CG:40	The elderly with RLS (EG: 69.34 ± 4.34; CG: 70.53 ± 5.51)	The group received routine care	Sleep quality	PSQI
Shamekh et al. (2022)	Egypt	Quasi‐experimental	(140 min) (1) (7) (20)	43–47 ≃ 45°C	EG:30	Pregnant women with RLS (EG: 25.90 ± 5.33)	Without control group	Sleep quality	GSQS

Abbreviations: CG, control group; DGSM, sleep and morning protocol by the german society of sleep research and sleep medicine; EG, experimental group; GSQS, groningen sleep quality scale; PSQI, petersburg sleep quality questionnaire; RLS, restless leg syndrome.

### 3.3 The Effect of Superficial Heat–Cold on the Sleep Quality of Patients With RLS

**FIGURE 2:**
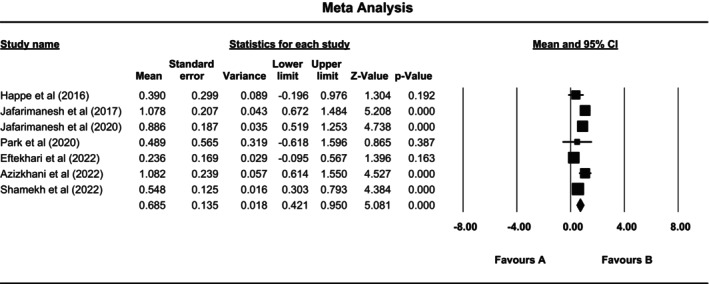
The effect of the superficial heat–cold application on the sleep quality of patients with RLS.

The results of the meta‐analysis in the present study indicated that superficial heat–cold application improved the sleep quality of patients with RLS (SMD = 0.685, 95% CI: 0.421 to 0.950). However, the results of studies by Happe et al. (SMD = 0.390, 95% CI: −0.197 to 0.977), Park et al. (SMD = 0.489, 95% CI: −0.617 to 1.596) and Eftekhari et al. (SMD = 0.236, 95% CI: −0.095 to 0.567) were not significant (Figure 2). Moreover, investigation revealed the heterogeneity of the studies (*I* ^2^ = 63.94%, *p* = 0.011).

### 3.4 Investigating the Source of Heterogeneity

In the present study, an attempt was made to investigate the source of heterogeneity using subgroup analysis and meta‐regression. In this regard and based on the results of subgroup analysis, the source of heterogeneity was not the study design, country or the control group.

**FIGURE 3:**
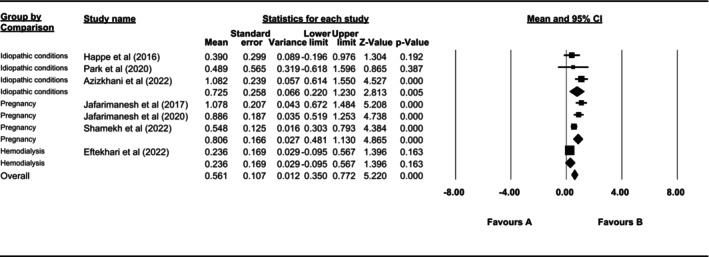
Subgroup analysis based on underlying clinical conditions.

Subgroup analysis based on underlying clinical conditions (including pregnancy, haemodialysis and lack of specific clinical conditions) in the subjects under study showed that studies with subjects who were pregnant (*I* ^2^ = 64.505%, *p* = 0.061) or had no underlying disease (*I* ^2^ = 43.872%, *p* = 0.168) were non‐heterogeneous. Investigating studies in terms of the total between‐group heterogeneity indicated that the studies were significantly heterogeneous between groups (*Q* = 7.441, *p* = 0.024); however, the total within‐group heterogeneity was not significant (*Q* = 9.198, *p* = 0.056) (Figure 3). The results also indicated that regarding underlying clinical conditions, the heat therapy was highly effective in pregnant women compared to other groups (SMD = 0.806, 95% CI: 0.481–1.130, *p* < 0.05).

**FIGURE 4:**
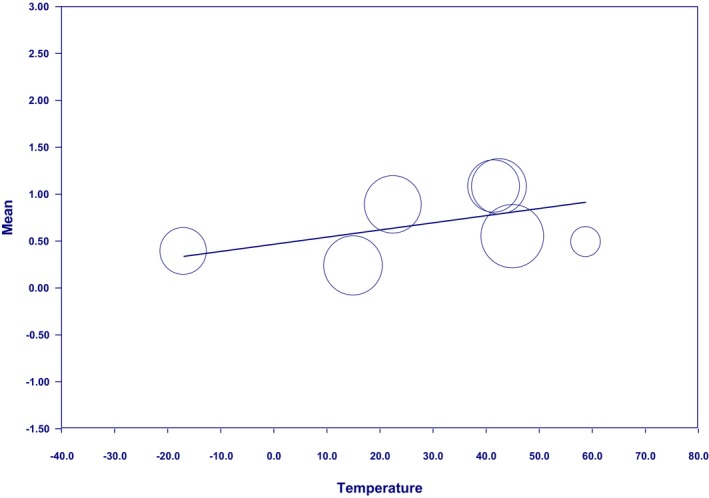
Meta‐regression based on intervention temperature.

In the present study and based on the meta‐regression results, it was revealed that as the temperature increased, the effectiveness of the intervention in improving sleep quality enhanced (*β* = 0.0182, 95% CI: 0.0096–0.0268, *p* < 0.05) (Figure 4). In this regard, the most remarkable improvement in the sleep quality of patients with RLS was achieved when the intervention temperature was 42.5°C.

**FIGURE 5:**
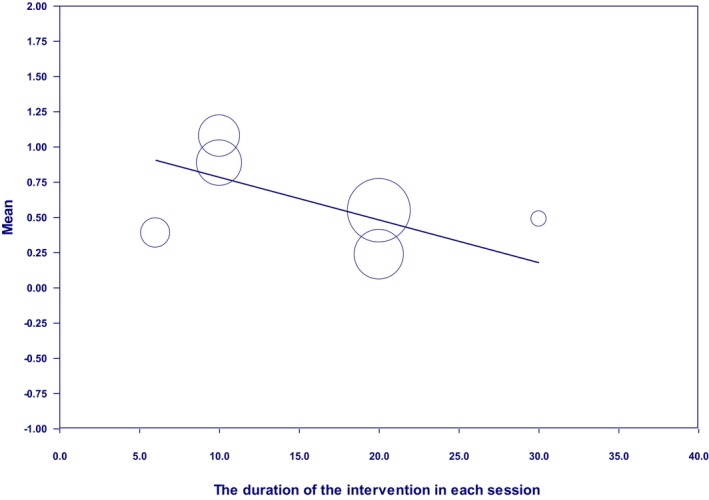
Meta‐regression based on the duration of the intervention in each session.

The results also showed a significant reduction in the effectiveness of the intervention in improving sleep quality with the increase in the intervention duration in each session (*β* = −0.031, 95% CI: −0.059 to −0.001, *p* < 0.05) (Figure 5). Accordingly, the greatest effectiveness was achieved when the duration of the intervention in each session was 10 min.

**FIGURE 6:**
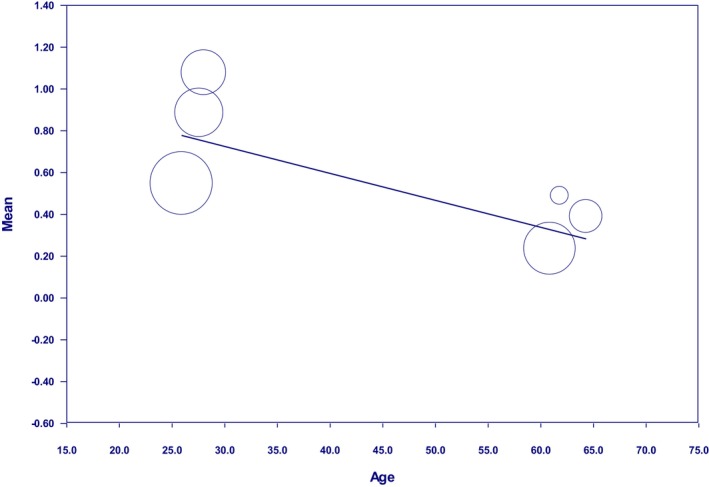
Age‐based meta‐regression in patients with RLS.

Other study results indicated that as the age of patients with RLS increased, the effectiveness of the intervention in improving their sleep quality decreased (*β* = −0.013, 95% CI: −0.024 to −0.001, *p* = 0.0259) (Figure 6). Consequently, the minor effectiveness occurred when the participants' age ranged between 60 and 65 years. The highest effectiveness was achieved when the participants were aged approximately between 20 and 25 years.

### 3.5 Publication Bias

**FIGURE 7:**
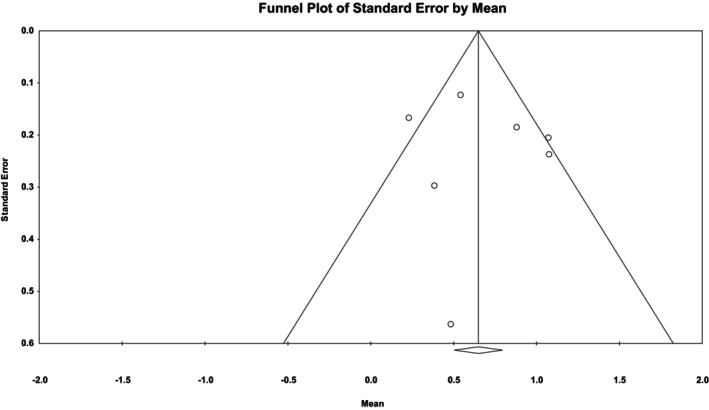
Publication bias.

In this research, the visual inspection of the publication bias using the funnel plot (Figure 7) and Egger's test results indicated no publication bias (Egger's test, *p* = 0.548).

### 3.6 Sensitivity Analysis

**FIGURE 8:**
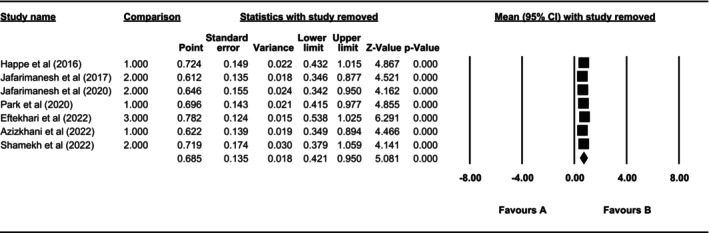
Examining the robustness of the results based on sensitivity analysis.

The robustness of the results was confirmed based on sensitivity analysis. A stable estimation of the total effect size in the meta‐analysis was obtained by excluding each study from the analysis. In other words, the sensitivity analysis showed that by excluding each study, the overall results did not change. Therefore, there is the robustness of the results in this study (Figure 8).

## 4 Discussion

The present study showed that superficial heat–cold application could improve the sleep quality of haemodialysis patients. This technique in medical history dates back to ancient times. For instance, Hippocrates, in his book ‘Management of Acute Disease’, recommended the use of hot water‐filled caps made of clay or metal for coastal joint pain (Porter 2001).

In addition to the present study, superficial heat–cold application has been emphasised as a beneficial intervention in other studies. Freiwald et al. (2021)'s study reported that superficial heat therapy could effectively manage low back pain. In this regard, several clinical trials have shown that heat therapy, alone or as part of a therapeutic approach, can lead to initial pain relief, improve muscle strength and flexibility, and facilitate patients' return to daily activities (Freiwald et al. 2018; Lewis et al. 2012; Petrofsky et al. 2015). Pain relief in patients with mild back pain is of great importance. In this regard, believe that heat therapy might eliminate the need for pain relievers, and in patients with moderate to severe pain, heat therapy may help reduce painkillers dosage Freiwald et al. (2021). Thus, superficial heat therapy is an efficient, safe, straightforward and cost‐effective non‐pharmacological pain relief alternative that patients can easily self‐administer. The present study and similar studies prove that treatment known for centuries still plays an essential role in clinical practice today (Freiwald et al. 2021; Stark et al. 2014).

The results of the present study provided settings based on which the effectiveness of the intervention in improving sleep quality can be improved. In this regard, these results indicated that by increasing the temperature of the intervention, its effectiveness in improving the sleep quality of patients with RLS enhances. This finding can be justified as the increase in temperature can lead to vasodilation, increase in capillary permeability, acceleration of cell metabolism, relaxation of muscles, acceleration of inflammation, soothing effects, reduction of synovial fluid viscosity to reduce joints stiffness, pain reduction by muscle relaxation and improved blood flow in foot cells (Freiwald et al. 2021; Petrofsky et al. 2015, 2016). Another noteworthy point was that, in this study, the optimum effectiveness in improving sleep quality was obtained at 42.5°C. In other words, according to the findings of the present study, from a nursing care point of view, if nurses employ a temperature of 42.5°C when applying superficial heat, compared to other temperatures, optimal effectiveness in improving the sleep quality of patients with RLS will be achieved.

Another finding of this research was that with an increase in the duration of the intervention in each session, contrary to the common belief, the effectiveness of the treatment did not increase. Accordingly, it was revealed in this study that the ultimate duration of the intervention in each session was 10 min. The logical justification of this finding seems perplexing; however, to further investigate this issue, it is recommended to conduct more original studies. In this regard, the present study's findings have indicated so far that the optimum effectiveness of the superficial heat–cold application is achieved if a higher temperature and shorter duration of the intervention in each session are implemented.

Other results of this study showed that the effectiveness of intervention in improving sleep quality decreases with the age of RLS patients. This finding can be justified based on two facts: there is a possibility that with the participants ageing, additional clinical problems appeared in them, affecting their sleep quality. In other words, older people struggle with diseases that challenge sleep quality management. Another fact is that based on the results of Gadie et al. (2017)'s study, as individuals age, their sleep quality decreases. Consequently, more impaired sleep quality is observed in older people, requiring stronger interventions to be improved. Based on the present study results, the highest effectiveness of the intervention was obtained when individuals were approximately in the age range of 20–25 years. Moreover, another factor in improving the effectiveness of the intervention was applying the intervention to younger individuals, which could achieve optimal results.

Another result was that the underlying clinical conditions in patients with RLS could be one of the sources of heterogeneity among the studies. In this regard, it was shown that compared to other clinical conditions, pregnant women received greater effectiveness of superficial heat–cold application, which is, conceivably, related to participants' age. In other words, women of reproductive age are customarily young, and the previous finding indicated that younger age could improve the effectiveness of the intervention. However, further investigation using original studies is necessary.

Fortunately, there was no publication bias in the present study, and the robustness of the results based on sensitivity analysis was confirmed. In other words, each study per se was not able to change the overall results, and the significance of the overall result was not affected by the results of one study.

The limitation of this study was the restricted number of original studies published in this field. It is recommended that more original studies in this field be published in the future so that the researchers will be granted the opportunity to update this study's results. In our study, we acknowledge the challenges associated with the quality of the available literature in our field of study. We emphasise the pressing need for further well‐designed studies to overcome these challenges. By doing so, the aim is to provide a balanced perspective on the subject matter to readers and to encourage future researchers to address the existing gaps in the literature.

## 5 Implications For Clinical Practice and Research

Nurses and health care providers can include this intervention in the care plan for patients with RLS. Using this cost‐effective method while considering the settings described, the optimum effectiveness of the intervention in improving the sleep quality of patients can be achieved.

## 6 Conclusion

The present study suggests that superficial heat–cold application may potentially improve the sleep quality of patients with RLS. Moreover, in this study, some settings were suggested according to which maximum effectiveness of the intervention could be achieved. The ultimate effectiveness was obtained when a higher temperature (42.5°C) was used during sessions in lower age groups. The present study provided some preliminary insights into the potential role of superficial heat–cold application as a nonpharmacological treatment option for RLS.

## Author Contributions

M.M.M., M.A. and A.A.V.R. conceptualised, analysed and interpreted the data, and drafted the manuscript. M.M.M. designed the study and participated in the analysis and interpretation of data. M.M.M. and A.A.V.R. coordinated the study, revised the manuscript, edited and approved the final version to be submitted for publication, and helped in the analysis and interpretation of data. All authors read and approved the final manuscript.

## Ethics Statement

The authors have nothing to report.

## Consent

The authors have nothing to report.

## Conflicts of Interest

The authors declare no conflicts of interest.

## Data Availability

The datasets used during the current study are available from the corresponding author on reasonable request.

## Appendix 1 Search Strategy Defined for Each Database

### 1.1

**Table:**

Databases	Search Strategies
PubMed	(“Cryotherapy”[MeSH Terms] OR “thermotherapy”[Title/Abstract] OR “temperature”[Title/Abstract] OR “heat wrap” [Title/Abstract] OR “cold wrap” OR “water bottles”[Title/Abstract] OR “stone therapy”[Title/Abstract] OR “heat pack”[Title/Abstract] OR “cold pack”[Title/Abstract] OR “hot poultices”[Title/Abstract] OR “cold poultices”[Title/Abstract] OR “heat lamp”[Title/Abstract] OR “electric heat pads”[Title/Abstract] OR “hydrotherapy”[Title/Abstract] OR “hot water baths”[Title/Abstract] OR “cold water baths”[Title/Abstract] OR stream[Title/Abstract] OR sauna[Title/Abstract]) AND (“Sleep quality”[MeSH Terms] OR “Sleep Initiation and Maintenance Disorders”[MeSH Terms] OR sleeplessness[All Fields] OR insomnia[All Fields] OR “sleep maintenance”[All Fields] OR “sleep disturbance”[All Fields] OR “sleep problem”[All Fields] OR sleep[Title/Abstract] OR “sleep promotion”[All Fields])
Scopus	(TITLE‐ABS‐KEY (Cryotherapy) OR TITLE‐ABS‐KEY (thermotherapy) OR TITLE‐ABS‐KEY (cold wrap) OR TITLE‐ABS‐KEY(temperature) OR TITLE‐ABS‐KEY(heat wrap) OR TITLE‐ABS‐KEY(water bottles) OR TITLE‐ABS‐KEY(stone therapy) OR TITLE‐ABS‐KEY(heat pack) OR TITLE‐ABS‐KEY(cold pack) OR TITLE‐ABS‐KEY(hot poultices) OR TITLE‐ABS‐KEY(cold poultices) OR TITLE‐ABS‐KEY(heat lamp) OR TITLE‐ABS‐KEY(electric heat pads) OR TITLE‐ABS‐KEY(hydrotherapy) OR TITLE‐ABS‐KEY(hot water baths) OR TITLE‐ABS‐KEY(cold water baths) OR TITLE‐ABS‐KEY(stream) OR TITLE‐ABS‐KEY(sauna)) AND (TITLE‐ABS‐KEY(Sleep quality) OR TITLE‐ABS‐KEY(Sleep Initiation and Maintenance Disorders) OR TITLE‐ABS‐KEY(sleeplessness) OR TITLE‐ABS‐KEY(insomnia) OR TITLE‐ABS‐KEY(sleep maintenance) OR TITLE‐ABS‐KEY(sleep disturbance) OR TITLE‐ABS‐KEY(sleep problem) OR TITLE‐ABS‐KEY(sleep promotion))
ISI	(TS = (Cryotherapy) OR TS = (thermotherapy) OR TS = (cold wrap) OR TS = (temperature) OR TS = (heat wrap) OR TS = (water bottles) OR TS = (stone therapy) OR TS = (heat pack) OR TS = (cold pack) OR TS = (hot poultices) OR TS = (cold poultices) OR TS = (heat lamp) OR TS = (electric heat pads) OR TS = (hydrotherapy) OR TS = (hot water baths) OR TS = (cold water baths) OR TS = (stream) OR TS = (sauna)) AND (TS = (Sleep quality) OR TS = (Sleep Initiation and Maintenance Disorders) OR TS = (sleeplessness) OR TS = (insomnia) OR TS = (sleep maintenance) OR TS = (sleep disturbance) OR TS = (sleep problem) OR TS = (sleep promotion))
ProQuest	(ti(Cryotherapy) OR ti(thermotherapy) OR ti(cold wrap) OR ti(temperature) OR ti(heat wrap) OR ti(water bottles) OR ti(heat pack) OR ti(cold pack) OR ti(hot poultices) OR ti(cold poultices) OR ti(heat lamp) OR ti(electric heat pads) OR ti(hydrotherapy) OR ti(hot water baths) OR ti(cold water baths) OR ti(stream) OR ti(sauna) OR ab(Cryotherapy) OR ab(thermotherapy) OR ab(cold wrap) OR ab(temperature) OR ab(heat wrap) OR ab(water bottles) OR ab(heat pack) OR ab(cold pack) OR ab(hot poultices) OR ab(cold poultices) OR ab(heat lamp) OR ab(electric heat pads) OR ab(hydrotherapy) OR ab(hot water baths) OR ab(cold water baths) OR ab(stream) OR ab(sauna)) AND (ti(Sleep quality) OR ti(Sleep Initiation and Maintenance Disorders) OR ti(sleeplessness) OR ti(insomnia) OR ti(sleep maintenance) OR ti(sleep disturbance) OR ti(sleep problem) OR ti(sleep promotion) OR ab(Sleep quality) OR ab(Sleep Initiation and Maintenance Disorders) OR ab(sleeplessness) OR ab(insomnia) OR ab(sleep maintenance) OR ab(sleep disturbance) OR ab(sleep problem) OR ab(sleep promotion))

## Appendix 2 Full‐Text Articles Excluded, With Reasons (*n* = 137)

### 2.1

Different study population (*n* = 90).

No intervention of interest (*n* = 29).

Not a RCT (*n* = 14).

No outcome of interest (*n* = 4).
